# Prevalence of dental caries in deciduous teeth and oral health related quality of life among preschool children aged 4–6 years in Kisarawe, Tanzania

**DOI:** 10.1186/s12903-020-1032-x

**Published:** 2020-02-10

**Authors:** Ray M. Masumo, Tumaini S. Ndekero, Lorna C. Carneiro

**Affiliations:** 1 Oral Health Section, Ministry of Health, Community Development, Gender, Elderly and Children, P. O. Box 743, Dodoma, Tanzania; 2 Department of Restorative Dentistry, Muhimbili University of Health and Allied Sciences, P.O. Box 65001, Dar es salaam, Tanzania

**Keywords:** Dental caries, Oral health related quality of life, Pre-school children

## Abstract

**Background:**

Preschool years are a critical period in the development of a healthy child. The consequences of poor oral health in preschool children reach beyond dental problems, with oral health-related quality of life (OHRQoL) being associated with overall systematic health as well as one’s quality of life. The purpose of this study was to assess the prevalence of dental caries and its impacts on the OHRQoL in a sample of preschool children in Kisarawe.

**Methods:**

A cross-sectional based study was conducted in 2017. A total of 1106 preschool children completed a face-to-face interview, using a translated Kiswahili version of the Michigan Oral Health-related Quality of Life Scale (MOHRQoL) –Child Version (2003), and underwent clinical oral examination using WHO (1997) criteria.

**Results:**

The decayed component was the most prevalent (dft = 2.08) and the Significant Caries Index (SiC) was 5.54 double of the (dft), showing polarization of dental caries in the studied population. After adjusting for appropriate covariates, preschool children of age 5 and 6 years old were more likely to have decayed tooth [Adjusted OR = 3.02, (95% CI =2.01–4.54)] and [Adjusted OR = 2.23, (95% CI = 1.55–3.20)] respectively. Preschool children without visible plaque on the buccal surface of upper anterior teeth were less likely to have decayed teeth [Adjusted OR = 0.21, (95% CI = 0.09–0.45)]. Regarding measurements of oral health-related quality of life using the MOHRQoL, only preschool children who reported on ‘do your teeth hurt you now?’ and ‘do kids make fun of your teeth?’ were more likely to have a decayed tooth [Adjusted OR = 1.74, (95% CI = 1.12–2.71)] and [Adjusted OR = 1.87, (95% CI = 1.11–3.15)], respectively.

**Conclusion:**

Findings from this study suggest that dental caries affects a significant portion of preschool children and, was associated with poor oral hygiene. The overall impacts of dental caries prevalence to OHRQoL were low in this sample of preschool children. Children having caries (independent variable) were shown to report more frequently that ‘do your teeth hurt you now?’ and ‘do kids make fun of your teeth?’ were more likely to have a decayed tooth among preschool children in Kisarawe, Tanzania.

## Background

Despite the decline in the prevalence of dental caries in children in western countries, caries in preschool children remains a major problem in both developed and developing countries [1]. Dental caries is a common infectious disease, where acid-producing bacteria, known as *Mutans Streptococci*, live in the tissues of the mouth and metabolize sugars. The acid, produced over time, demineralizes the tooth structure and causes caries [1, 2]. Sugary food and beverage consumption have a major impact on an individual’s experience of caries, both in childhood and in adulthood. High sugar intakes increase the amount of *Mutans Streptococci* in the mouth, which in turn increases the chance of destruction of teeth [1, 2]. Previous research has identified a range of risk factors for developing dental caries to preschool children that involves a complex interaction of biological, social and economic factors, not dissimilar to factors that increase the susceptibility of developing caries at later life stages [1–3].

Preschool years are a critical period in the development of a healthy child [1, 3, 4]. In many developing countries, over 90% of dental caries in preschool children has remained untreated [1–3]. The consequences of poor oral health to preschool children reach beyond dental problems, with oral health being associated with overall systematic health as well as one’s quality of life [5]. In 1992, Acs and colleagues conducted a study of three-year-old children and reported that preschool on average children with decayed teeth weighed one kilogram less than children without decayed teeth [6]. In another study, Ayhan and colleagues in 1996 reported a similar finding that among Turkish preschool children between ages 3 to 5 years, children with decayed teeth were significantly lighter and shorter than preschool children without caries [7]. The oral health status of preschoolers relies heavily on the caregivers; therefore, understanding what parents and teachers know about oral health is crucial when working towards modifying behaviors and encouraging health promotion [1, 2, 8].

Untreated caries significantly impacts on the quality of life of preschool children and their dietary intake [1, 9]. OHRQoL is a multidimensional construct that includes a subjective evaluation of the individual’s oral health, functional well-being, emotional wellbeing, expectations and satisfaction with care, and sense of self [10, 11]. OHRQoL concept fulfills the framework of patient assessment, as the World Health Organization (WHO) defines quality of life as individuals’ “perceptions of their position in life in the context of culture and value systems in which they live, and in relation to their goals, expectations, standards, and concerns” [10–13].

There is strong evidence that demonstrates children with untreated decayed teeth had significantly poorer oral health-related quality of life (OHRQoL) than children without decayed teeth as assessed both by the children and their parents [14, 15]. Other studies have shown that preschool children with a decayed tooth have a higher risk of increased days with restricted activity and absence from school, and a diminished ability to learn [16]. Further, caries in preschool children has an impact not only on the child’s educational development, but also on the economy of the family due to time taken off work by parents in order to take children to health care facilities [17]. Moreover, research has shown that parents’ lack of knowledge and negative attitudes towards preschool oral health care or interventions are strongly associated with an increased caries experience in preschoolers [7].

There are a growing number of studies on dental caries status and OHRQoL in many countries, especially industrialized countries [11], however, published research regarding preschool children’s dental caries status and OHRQoL in Tanzania is minimal. While the results from studies based in other countries provide relevant information related to this subject [11], but these results cannot be entirely relatable to the preschool children in Tanzania.

In 1991, Kerosuo and Honkala conducted a population-based study among preschool children in Dar es Salaam, Tanzania, and Hyvinkaa, Finland [18]. In all age groups, the Tanzanian children had higher caries experience than Finnish children. In the Finnish group, caries experience was higher among 7 years old than 3 years old, while the corresponding findings in Tanzanian group, no statistically significant difference was found between age groups [18]. In both groups, maxillary incisors and molars were the teeth most frequently affected by caries. Frequent consumption of sweet snacks and drinks increased the risk for caries in Tanzanian but not in Finnish children. High socioeconomic status decreased the risk for caries in Finnish children but among Tanzanian children high socioeconomic status was not associated significantly with the risk of developing caries [18]. This research work [18] was probably performed in the late 1980s and in the meantime the dietary habits and maybe socioeconomic status has changed. In another study, Matee and colleagues recruited children ages 1 to 4 from nine regions of Tanzania mainland, and reported prevalence varied from 1.5% in Dodoma to 12.8% in Morogoro (rural) [19]. In 2017, Mwakayoka and colleagues conducted a study in urban and peri-urban areas of the Mbeya region and reported a caries prevalence of 8.4 and 20.6% among 2-year-old and 3 to 4-year-old children, respectively. Evidence from studies conducted in Tanzania among preschool children has shown that children with poor oral hygiene had a statistically significantly higher prevalence of dental caries than their counterparts [20].

### Purpose

The purpose of this study was to assess the prevalence of dental caries and its impacts on the oral health-related quality of life (OHRQoL) in a sample of preschool children aged 4–6 years in Kisarawe, Tanzania. Detailed information regarding the prevalence of dental caries and its impacts on OHRQoL provides a valuable tool in the planning, implementation and evaluation of oral health promotion programs. Such evidence is rare when it comes to preschool children in Tanzania.

## Method

The present paper is based on data generated from a cross-sectional study conducted in Kisarawe district, one of the 6 districts in the Coastal Region of Tanzania. It is administratively divided into 15 wards which have a semi–urban rural population: Cholesamvula, Kibuta, Kiluvya, Kisarawe, Kurui, Mafizi, Maneromango, Marui, Marumbo, Masaki, Msanga, Msimbu, Mzenga, Vihingo, and Vikumbulu. The district is home to about 101,598 people, out of whom 2.77% is 4 years old, 2.81% is 5 years old and 2.83% is 6 years old [21] and, had eighty-three registered preschools at the time of the study. The estimated sample size was calculated by assuming that the prevalence of dental caries in preschool children was 50%, with a margin error of 5%, confidence level of 95%, a power of 90% and an assumed design effect of 2. Another 5% was added to the sample size to account for non-responses. A sample size (*n* = 1106) of this magnitude is sufficient to the pre-calculated sample size of 810 preschool children.

Kisarawe district was conveniently chosen due to its rural (population of 84,174) and semi-urban (population of 17,424) characteristics [21]. The structure of the formal education and training system in Tanzania constitutes of 2 years of pre-primary education, 7 years of primary education, 4 years of secondary (Ordinary Level), 2 years of secondary (Advanced Level), and a minimum of 3 years of university education [22]. Official school age of pre-primary education is 4–6 years [22]. One preschool from each ward was randomly selected to participate in this study. Head-teachers of the thirty-one selected schools were informed of the study and requested to provide each child in the register a consent form for parents/guardians to sign. Children involved in the survey were all in education and given consent to participate via a Term of Consent signed by parents or guardians allowing the clinical examination. This study was approved by the Medical Research Coordinating Committee of Muhimbili University of Health and Allied Sciences in Tanzania Ref. No. DA282/298/01.C.

### Interviews

An interview schedule was constructed in English and translated into Kiswahili, the main language in Kisarawe. Kiswahili is the national language in Tanzania spoken proficiently by almost 99% of the population. The interview schedule was translated in several steps; from English into Kiswahili by bi-lingual Kiswahili/English professionals, and then translated back to English by independent translators. Project professionals in the field reviewed the interview schedule for semantic, experiential and conceptual equivalence to the original version. Sensitivity to culture and selection of appropriate words were considered. The interview schedule was piloted and administered face-to-face before the children underwent a full oral clinical examination. Demographic characteristics were assessed in terms of preschool children’s age and sex. As shown in Table 1, the Michigan Oral Health-related Quality of Life Scale (MOHRQoL) –Child Version, adapted from the work of Filstrup and colleagues [14], guided the selection of oral health-related quality of life variables and the multivariable analyses. The Michigan Oral Health-Related Quality of Life Questionnaire utilizes multidimensional scales to evaluate the OHRQoL of children [14]. It is multidimensional because it includes items such as functional, social, and psychological aspects. This questionnaire consists of child and parent versions [14]. It is intended for children 4 years of age and older. The child version contains items that were distributed throughout 3 areas, including pain, functional, and psychological aspects. Each interview of a preschool child was conducted in a private, quiet place outside the classroom and Yes = 1 and No = 0 responses to questions were noted.

**Table 1:** Michigan Oral Health-related Quality of Life Scale–Child Version (Filstrup et al. [14])

Screening questions for preschoolers	
1. Do your teeth hurt you now?	
2. Do your teeth hurt when you eat something hot or cold?	
3. Do your teeth hurt when you eat something sweet?	
4. Does a hurting tooth wake you up at night?	
5. Does a hurting tooth stop you from playing?	
6. Is it hard for you to chew and bite?	
7. Do you like your teeth?	
8. Are you happy with your teeth and smile?	
9. Do kids make fun of your teeth?	

Parents of preschoolers were also invited to participate, but too few parents were present on interview day so was not enough to include them in the final analysis. Therefore parent/proxy measure was not the part of this study.

### Clinical examination

Clinical oral examinations were conducted by trained and calibrated dentists (TN and LC), whereas trained assistants recorded the observations. Calibration exercises for the examiners with respect to childhood caries were carried out according to the guidelines published by the British Association of the Study of Community Dentistry (BASCD) [23]. Children were examined in knee-to-knee position using a dental mirror and natural light. Current oral hygiene in terms of visible plaque in the upper anterior teeth, central and lateral incisors (52, 51, 61, 62) and also canine (53. 63) each tooth was recorded as (0) No or (1) Yes. Dummy variables (0 = No, 1 = Yes) were summarized (range 0–6) and dichotomized into children with a count of a plaque score of 0 as having “good oral hygiene” while children with plaque score of one or more were regarded as having “poor oral hygiene”. Teeth were cleaned and dried by sterile gauze and inspected for dental caries using disposable dental mirrors. Dental caries status was accessed using the criteria recommended by the World Health Organization (WHO), Oral health surveys: Basic methods, 1997 [24]. Caries was limited to deciduous teeth and was diagnosed at cavitation level mainly by visual inspection and no radiographs were taken, and the presence of a carious lesion was scored = 1, while absence of any visible lesion was scored = 0. Those with a decayed component > 0 were regarded as having dental caries while those who had a decayed component = 0 were caries-free. The dmft was reformatted to dft to avoid confusion of recording deciduous missing teeth which might be due to caries or exfoliation. There were no teeth filled. In the current analysis, decayed teeth (dft) was used as dependent variable, both dichotomized as (0) absent (dft = 0) and (1) present (dft =1) and used as a count variable.

### Statistical analyses

Predictive Analytics Software, IBM SPSS Statistics, version 20 was used for data analysis. Univariate analyses were performed by use of chi-square statistics. A probability value of *p* < 0.05 was considered statistically significant. Multiple variable analyses were performed using logistic regression with odds ratios (OR) and 95% confidence intervals (CI) and Poisson regression with rate ratios (RR). Since using dummy variables runs the risk of losing information, results from logistic regression analyses were checked using Poisson regression with count variable were used to identify adjusted independent variables; demographic (age and sex), oral hygiene (plaque status) and measurement of OHRQoL by the Michigan Oral Health-related Quality of Life Scales (MOHRQoL) –children Version.

## Results

### Sample characteristics and descriptive analyses

**Fig. 1:**
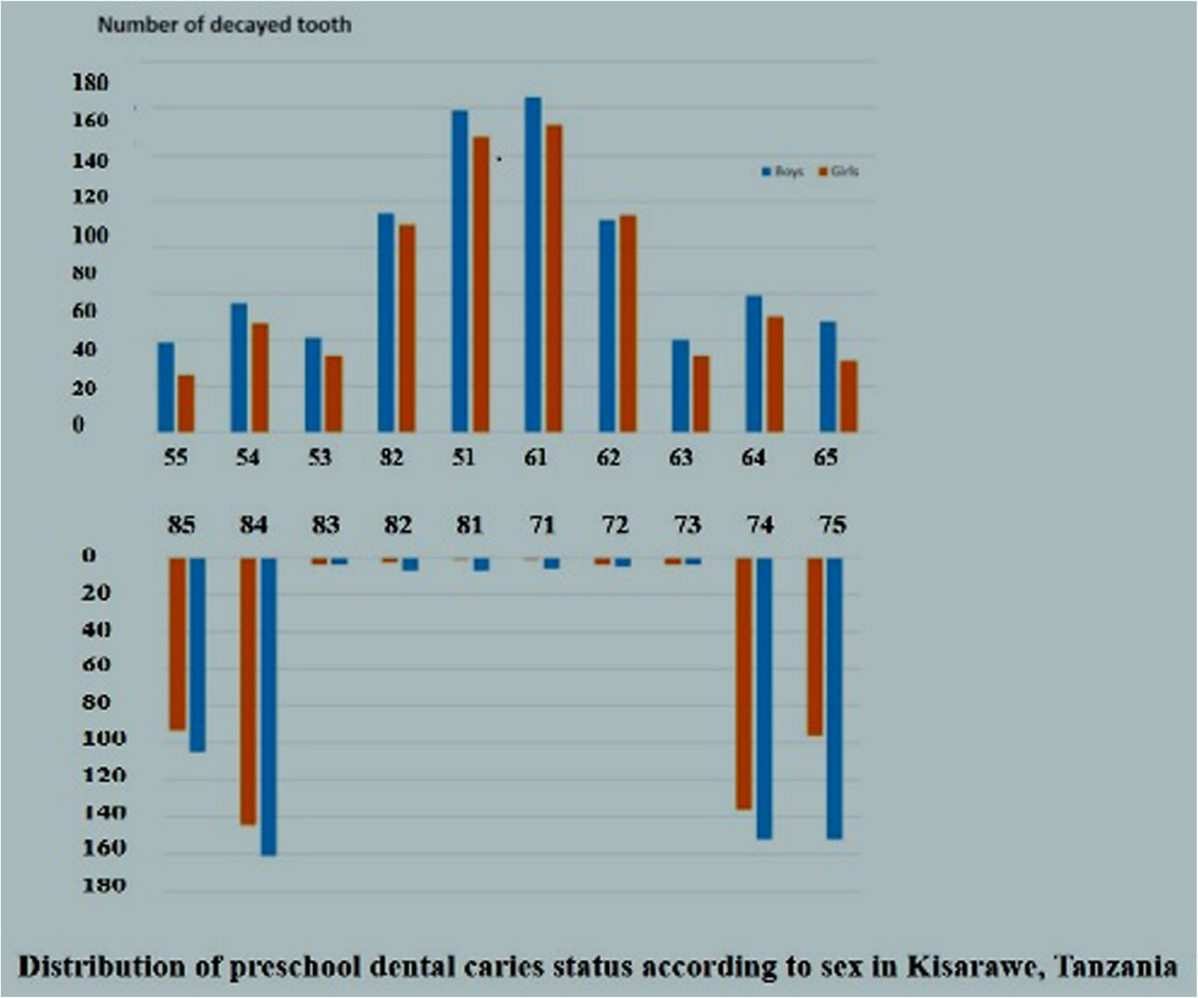
Distribution of preschool dental caries status accordong to sex in Kisaware, Tanzania

**Fig. 2:**
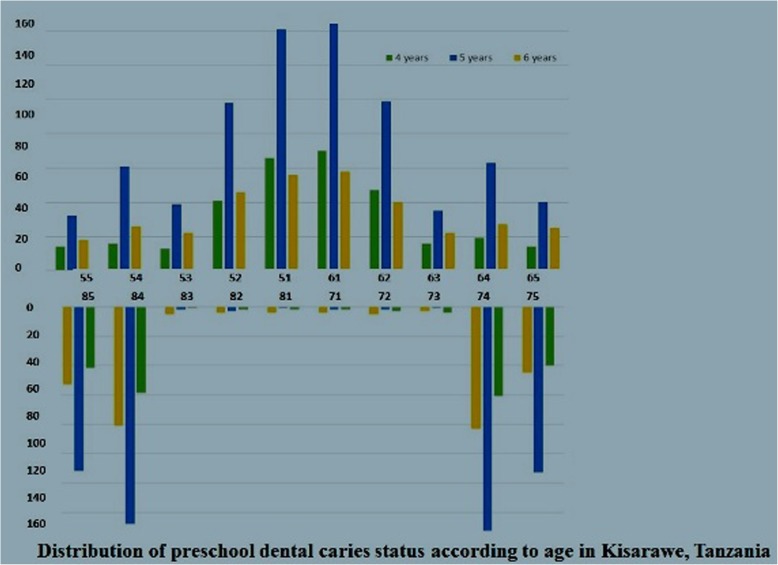
Distributiom of preschool dental carries status according to age in Kisaware, Tanzania

Out of 1132 preschool children approached, 1106 agreed to participate, been 270 children of 4 years old (24.4%), 562 children of 5 years old (50.8%) and 274 children of 6 years of age (24.8%) corresponding to a response rate of 97.7%. Totals of 49.9% of the children investigated were females and the mean age was 4.97 years (SD 0.93). The decayed component was the most prevalent (dft = 2.08) and Significant Caries Index (SiC) was 5.54 double of the dft, showing polarization of dental caries in the studied population. As shown in Fig. 1, the tooth specific pattern of caries was similar across preschool boys and girls in the study site. Maxillary central incisors and mandibular molar teeth were the most affected teeth both in preschool boys and girls. The frequency distribution of caries experience according to tooth type and sex showed that boys had consistently higher caries experience than girls across all teeth.. However, the caries rates were highest among 5 years preschool children for each tooth type as depicted in Fig. 2. This could be explained because of irreversibility of dentine lesions and/or shedding of primary molars, As shown in Table 2 the frequency distribution of participants by dental caries experiences status (dependent variable) by demographic (age and sex), current oral hygiene status (visible plaque status) and measurement of OHRQoL by the Michigan Oral Health-related QOL Scales–children Version (independent variables). About one third (30.2%) of participating preschool children had caries experience, whereas nearly all (93.2%) of preschool children had visible plaque on buccal surfaces of upper anterior teeth. Twenty three percent of preschool children reported experience of tooth pain while less than 20 % (18.5%) reported experience of pain on eating hot or cold. It was also found that majority (97.6%) of the preschool children reported to like their teeth, 98.2% were happy with appearance of their teeth and they can smile freely and only 6.3% reported that other kids made fun of their teeth.

**Table 2:** Frequency distribution of study population attributes (*n* = 1106)

No	Variable	Categories	% (n)
1	Age	4 yrs	24.4 (270)
		5 yrs	50.8 (562)
		6 yrs	24.8 (274)
2	Sex	Male	50.1 (554)
		Female	49.9 (552)
3	Decayed teeth	At least one tooth decay	30.2 (334)
		No teeth decay	69.8 (772)
4	Visible plaque	Yes	93.2 (1031)
		No	6.8 (75)
5	Does your teeth hurt you now?	Yes	22.9 (253)
		No	77.1 (853)
6	Does your tooth hurt when you eat something hot or cold?	Yes	18.5 (205)
		No	81.5 (901)
7	Does your tooth hurt when you eat something sweet?	Yes	18.4 (203)
		No	81.6 (903)
8	Does a hurting tooth wake you up at night?	Yes	14.4 (159)
		No	85.6 (947)
9	Does a hurting tooth stop you from playing?	Yes	12.6 (139)
		No	87.4 (967)
10	Is it hard for you to chew and bite?	Yes	19.2 (212)
		No	80.8 (894)
11	Do you like your teeth?	Yes	97.6 (1080)
		No	2.4 (26)
12	Are you happy with your teeth and smile?	Yes	98.2 (1086)
		No	1.8 (20)
13	Do kids make fun of your teeth?	Yes	6.3 (70)
		No	93.7 (1036)

### Reliability and frequency of preschool children dental caries

Clinical oral examination of preschoolers was carried out by two trained and calibrated dentists (TN and LC). Prior to the fieldwork, the calibration for scoring preschool dental caries was conducted with the examiners, who underwent a training exercise for the diagnosis of preschooler dental caries. Calibration was performed using images of different clinical situations on two separate occasions with a one-week interval between sessions. The minimum intra-examiner agreement was 0.83 and the minimum inter-examiner agreement was 0.78. During the fieldwork, duplicate examinations one week apart were performed with 74 preschool children randomly chosen. Intra-examiner reliability in terms of Cohen’s kappa ranged from 0.81 to 0.89, respectively. Test re-test reliability was not performed for measurement of preschool children responses to the Michigan Oral Health-related Quality of Life (MOHRQoL) –children Version*.* The Pearson correlation coefficient for dft index (0–11) and the Kiswahili version of MOHRQoL (0–9) was 0.152, which is significant (*P* < 0.001 for a two paired test), based on 1106 complete observation (i.e. cases with non-missing value for both dft index and MOHRQoL index). The direction of this relationship is positive (i.e. dft index and MOHRQoL index are positively correlated) meaning that these variables tends to increase together (i.e. greater dft index is associated with greater MOHRQoL index), however the strength or effect size of this association is small (Correlation coefficients between 0.10 and 0.29 represent a small association). As shown in Table 3, preschool children dental caries was significant, and it increased with age. Of the 334 children who experienced caries, 100 were children 4 years of age (37%), 184 were children 5 years of age (32.7%) and 50 were children 6 years of age (18.2%) [χ^2^ = 26.276; *p* < 0.05]. The majority of preschool children with dental caries had visible plaque on the buccal surface of upper anterior teeth as compared to those without visible plaque [χ^2^ = 14.562; *p* < 0.05]. Regarding measurements of oral health-related quality of life (OHRQoL) using a modified version of Michigan Oral Health-related Quality of Life Scale–Child Version, preschool children with a decayed tooth reported pain [χ^2^ = 14.709; *p* < 0.05] and waking up at night because of pain [χ^2^ = 8.902; *p* < 0.05].

**Table 3:** Distribution of dental caries status of preschool children aged 4–6 years, univariate analysis (*n* = 1106)

No	Variable	Categories	% (n)	Chi square	*P*-value
1	Age	4 yrs	37 (100)	26.276	0.001
		5 yrs	32.7 (184)		
		6 yrs	18.2 (50)		
2	Sex	Male	31.6 (175)	1.017	0.313
		Female	28.8 (159)		
3	Visible plaque	Present	10.7 (8)	14.562	0.001
		Absent	31.6 (326)		
4	Does your teeth hurt you now?	Yes	39.9 (101)	14.709	0.001
		No	27.3 (233)		
5	Does your teeth hurt when you eat something hot or cold?	Yes	38.5 (79)	8.299	0.004
		No	28.3 (255)		
6	Does your teeth hurt when you eat something sweet?	Yes	38.9 (79)	8.964	0.003
		No	28.2 (255)		
7	Does a hurting tooth wake you up at night?	Yes	40.3 (64)	8.902	0.003
		No	28.5 (270)		
8	Does a hurting tooth stop you from playing?	Yes	36.0 (50)	2.513	0.113
		No	29.4 (284)		
9	Is it hard for you to chew and bite?	Yes	38.2 (81)	10.422	0.005
		No	28.2 (252)		
10	Do you like your teeth?	Yes	30.3 (327)	0.136	0.713
		No	26.7 (7)		
11	Are you happy with your teeth and smile?	Yes	30.5 (331)	2.232	0.135
		No	15.0 (3)		
12	Do kids make fun of your teeth?	Yes	47.1 (33)	10.178	0.001
		No	29.1 (301)		

### Adjusted factors for preschool children dental caries

All variables of socio-demographic (age)-, oral hygiene behavioral (visible plaque on the buccal surface of upper anterior teeth)-, and measurements of a Kiswahili version of Michigan Oral Health-related Quality of Life Scale–Child Version, that were statistically significantly associated with preschool dental caries status in univariate analyses (*P* < 0.05) were included in multiple variable logistic regression analyses and Poisson regression analyses. As depicted in Table 4, Multivariate logistic regression assisted us gain a deeper understanding of the relationship between independent variables and dental caries status of preschool children. The final logistic regression model revealed that, preschool children of age 5 and 6 years old were more likely to have decayed tooth [Adjusted OR = 3.02, (95% CI =2.01–4.54)] and [Adjusted OR = 2.23, (95% CI = 1.55–3.20)] respectively. Preschool without visible plaque in buccal surface of upper anterior teeth were less likely to have decayed teeth [Adjusted OR = 0.21, (95% CI = 0.09–0.45)]. Regarding measurements of oral health-related quality of life (OHRQoL) using a modified version of Michigan Oral Health-related Quality of Life Scale–Child Version, only preschool children who reported YES on ‘does your teeth hurt you now?’ and ‘do kids make fun of your teeth?’ were more likely to have a decayed tooth [Adjusted OR = 1.74, (95% CI = 1.12–2.71)] and [Adjusted OR = 1.87, (95% CI = 1.11–3.15)], respectively. Poisson regression confirmed the results from multiple variable logistic regression analysis presented in Table 4.

**Table 4:** Distribution of preschool dental caries status by socio-demographic (age), behavioral factor (current oral hygiene- visible plaque) and measurements of oral health-related quality of life (MOHRQoL, *n* = 1106)

No	Variable	Categories	% (n)	Adjusted OR; 95% CI	*P*-Value	Adjusted RR; 95% CI	*P*-Value
1	Age	4 yrs	37 (100)	1		1	
		5 yrs	32.7 (184)	3.02 (2.01–4.54)	0.000	2.38 (1.99–2.85)	0.000
		6 yrs	18.2 (50)	2.23 (1.55–3.20)	0.000	1.77 (1.50–2.09)	0.000
2	Visible plaque	Present	10.7 (8)	1		1	
		Absent	31.6 (326)	0.21 (0.09–0.45)	0.000	0.24 (0.16–0.36)	0.000
3	Does your teeth hurt you now?	No	39.9 (101)	1		1	
		Yes	27.3 (233)	1.74 (1.12–2.71)	0.014	1.66 (1.40–1.98)	0.000
4	Does your teeth hurt when you eat something hot or cold?	No	38.5 (79)	1		1	
		Yes	28.3 (255)	0.89 (0.50–1.58)	0.704	0.86 (0.69–1.09)	0.227
5	Does your teeth hurt when you eat something sweet?	No	38.9 (79)	1		1	
		Yes	28.2 (255)	0.91 (0.50–1.63)	0.754	1.07 (0.84–1.35)	0.569
6	Does a hurting tooth wake you up at night?	No	40.3 (64)	1		1	
		Yes	28.5 (270)	1.41 (0.87–2.30)	0.158	1.30 (1.08–1.56)	0.006
7	Do kids make fun of your teeth?	No	47.1 (33)	1		1	
		Yes	29.1 (301)	1.87 (1.11–3.15)	0.018	1.27 (1.05–1.55)	0.014

## Discussion

The purpose of this study was to explore the level to which dental caries impacts the oral health-related quality of life (OHRQoL) in a sample of preschool children in Kisarawe Tanzania.

This is one of the first population-based studies to systematically investigate the correlates of dental caries and oral health-related quality of life of preschool children in Kisarawe, Tanzania. Thus, this study provides information about preschool children that have not been well-covered by the national oral health survey in Tanzania. Information about the prevalence of dental caries in preschool children in sub-Saharan Africa is scarce, and the Kisarawe district has been surveyed to a very limited extent. There is a large body of literature that highlighted the role that individual macro factors such as socioeconomic and contextual factors such as ability to access, affordability and use of oral health services and proximal factors such as eating high sugar sugary food and beverages play a vital role on determinants of oral health [1, 9, 11, 25]. Therefore individual macro factors, the contextual factors and proximal factors such as consumption of sugary foods and beverages were not evaluated in this study. Other limitation factors on this study relate to the sample size and these results cannot be extrapolated to represent data for the whole population. This is due to the sample calculation being drawn from a specific population (preschool children enrolled in public schools). High response rates and a limited number of missing items in the interview, however, suggest that the study group, for whom there are complete data, reflects preschool children (4–6 yrs.) living in the catchment areas of the public schools in Kisarawe districts.

In terms of child-attributed factors, the prevalence of preschooler dental caries was associated with age and oral hygiene. A tendency was found regarding a greater prevalence of preschooler dental caries associated with an increase in age, and with caries being more common among the six-year-olds, in agreement with findings reported in previous studies [1–3]. This result can be explained that dental caries is a multifactorial chronic condition that requires time to develop and to be clinically detectable. So, the increase in the burden of dental caries disease due to age may be due by this and also, change in the dietary habits and hygiene practices in older children [1, 2].

Dental caries is highlighted as one of the most common diseases in children and adults and a serious public health problem. The identification of groups at risk for disease development therefore presents fundamental importance for its prevention and early treatment. In the present study, a percentage of caries-free preschool children of 69.8% was found; this result was similar to the observed 59% among preschool children in Abu Dhabi, United Arab Emirates [26] and 51% among preschool children in Hong Kong [27]. However, the results found in this research were higher than the 80% of children who are caries-free children reported in Mbeya Tanzania [20]. Differences in the reported prevalence of dental caries could be caused by the materials and methods employed in the different researches, study age groups or it could be a fact that preschool dental caries was prevalent in this study population.

Poor oral hygiene is one of the risk factors of preschooler’s dental caries [1, 3]. The access of preschool children to different kind of toothbrushes (modern and traditional such as miswaki) and also, the use of fluoridated toothpaste were not evaluated in this study. The majority of the children in the present study exhibited poor oral hygiene, characterized by the presence of clinically visible plaque [93.2% (*n* = 1031)]. Studies have documented an association between dental caries and tooth brushing supervision, and concluded that preschoolers do not yet have the manual dexterity needed for the maintenance of adequate oral hygiene [1–3]. Consistent with international evidence, the present study demonstrated that preschoolers who had absent visible plaque were less likely to develop dental caries [Adjusted OR = 0.21, (95% CI = 0.09–0.45)]. As mothers or caregivers’ supervision during tooth brushing of preschoolers was not part of the present study, the high prevalence of unsatisfactory oral hygiene may be explained by a lack of supervision of mothers or caregivers during tooth brushing, or else a lack of knowledge among parents regarding adequate oral hygiene practices [2, 3].

Several instruments have been proposed to measure children’s quality of life and should be selected depending on the desired outcome and characteristics of the target population. These instruments should be easy to understand, have questions that are short, clear, simple, relevant to the objectives of the study, and previously validated and it should be noted that quality of life is a construct and cannot be directly measured [8]. They include the Child Perceptions Questionnaire [28], the Child Oral Impacts on Daily Performances Index [29], the Child Oral Health Impact Profile [30], the Early Child Oral Health Impact Scale [15] and the Scale of Oral Health Outcomes for 5-year-old children [31], the Michigan Oral Health-Related Quality of Life scale [14] and the Pediatric Oral Health Related Quality of Life Measure [32]. All but the Michigan Oral Health-Related Quality of Life scale [14] and Early Child Oral Health Impact Scale [15] were designed for self-report. The Michigan Oral Health-Related Quality of Life scale, MOHRQoL [14] was chosen as the objectives of this study were to assess the effects of dental caries on oral health-related quality of life as reported by the children themselves of 4 years and above.

Studies have documented that, when possible, both child and parents should be asked to provide ratings of OHRQoL in an effort to provide a more well-rounded depiction of the child’s oral health care needs and quality of life issues [33]. Even if child’s opinion is the most valuable, there are certain factors which may compromise the reliability and validity of a child’s OHRQoL responses. Some of these factors include: short-term memory, a strong influence of recent incidents, lack of a fully developed long-term perspective, language problems during interviews, and reading problems when completing a written questionnaire [33].

In contrast to findings from this study that found fewer children with dental caries reported to be in pain, other studies report that the severity of dental caries has a negative influence on a child’s OHRQoL [34]. This could be due to the fact that the acute stage in caries is cyclic in nature as a carious tooth may have become necrotic or created a fistula through the bone relieving the pressure and pain. It is also possible that these children have experienced chronic pain and may describe a tooth that is only slightly uncomfortable as not painful or that their tolerance to pain is high.

Similar to findings of this study, the most frequently reported impacts were ‘pain in the teeth, mouth, or jaws’ [35] and the associated pain from dental caries has a negative impact on children’s emotional status, sleep patterns, and ability to learn or perform their usual activities [2]. Another study conducted among children and adolescents found a high dental caries experience and that dental caries had a negative impact on OHRQoL [12]. From the child’s perspective, the sequela of dental caries could have been transient and that on the day of the interview the tooth no longer hurt. Or it is possible that the child felt that a tooth that spontaneously hurts throughout the day and/or night was worse than eating. The reported pain to the different questions indicates that dental caries goes through different stages.

Contrastingly to findings from this study other studies observed that the prevalence of having an impact of dental caries was almost three times higher for children with dental caries with negative impacts on items related to pain, and to difficulty drinking and eating some foods [13]. In addition, another study found that an increase in the severity of early childhood caries resulted in the child’s having an impaired quality of life [36]. The relatively low number of OHRQoL impacts found in this study can be attributed to the sample’s community-based nature and young age. To our knowledge in Tanzania, only the Masumo and colleagues [15] has examined OHRQoL using Early Child Oral Health Impact Scale (ECOHIS) among infants and toddlers to-date. Taking OHRQoL impacts into account, however, can differentiate needs and help prioritize care for vulnerable populations [29]. This information is important as most studies indicate a modest yet significant correlation between unmet needs like dental decay and children’s OHRQoL.

## Conclusion

In conclusion, the findings of the present study suggest that dental caries affects a significant portion of preschool children in Kisarawe district Tanzania and, was associated with poor oral hygiene. The overall impacts dental caries prevalence to OHRQoL were low in this sample of preschool children. Children having a decayed tooth more frequently reported on ‘do your teeth hurt you now?’ and ‘do kids make fun of your teeth?’ were more likely to have a decayed tooth. Future studies should assess risk indicators using longitudinal analyses. Detailed information regarding the prevalence of dental caries and its impacts on OHRQoL provides a valuable tool in the planning, implementation and evaluation of oral health promotion programs. Such evidence is rare when it comes to preschool children in Tanzania.

## Data Availability

The datasets used and/or analysed during the current study available from the corresponding author on reasonable request.

## Abbreviations

BASCD: British Association of Community Dentistry
ECOHIS: Early Child Oral Health Impact Scale
MOHRQoL: Michigan Oral Health Related Quality of Life
OHRQoL: Oral Health Related Quality of Life
WHO: World Health Organization
